# Brief adult respiratory system health status scale-community version (BARSHSS-CV): developing and evaluating the reliability and validity

**DOI:** 10.1186/s12913-018-3505-z

**Published:** 2018-09-03

**Authors:** Hongzhe Dou, Yuejia Zhao, Yanhong Chen, Qingchun Zhao, Bo Xiao, Yan Wang, Yonghe Zhang, Zhiguo Chen, Jie Guo, Lingwei Tao

**Affiliations:** 1 Affiliated Hospital of Hebei University, No.212 Yuhua East Road, Baoding, 071000 China; 2 The NO.5 Hospital of Baoding, No.340 Ruixiang Street, Baoding, 071000 China; 3 College of Nursing, Hebei University, No.342 Yuhua East Road, Baoding, 071000 China; 4 School of Public Health, Capital Medical University, No.10 Xitoutiao, Youanmenwai, Beijing, 100069 China

**Keywords:** Community, Adult, Respiratory system, Health level, Reliability, Validity

## Abstract

**Background:**

The evaluation of respiratory system health status in hospitalized patients is usually based on many laboratory examinations and imaging examinations. Medical examinations require a lot of manpower, material resources, financial resources, and may cause a certain degree of mechanical damage and radiation damage. It is not easily used widely and economically to assess the respiratory health status of community adults. Therefore, researchers developed a brief adult respiratory system health status scale-community version (BARSHSS-CV) and tested its reliability and validity.

**Methods:**

Using clinical characteristics and pathogenic factors of respiratory system diseases as a theoretical basis and through reference to relevant literature, researchers developed an initial scale. A randomized cluster sampling strategy was used to recruit adults in the communities of Baoding City, Shijiazhuang City, Cangzhou city and Chifeng City in China. Researchers randomly selected 1 district from each city. Subsequently, 4 communities were respectively randomly selected from 4 districts. Then, researchers conducted the questionnaire survey in 4 communities. Finally, researchers investigated 615 community adults. 584 valid questionnaires were recovered. By applying exploratory factor analysis, confirmatory factor analysis, content validity index, Cronbach’s α coefficient, mean inter-item correlation coefficient and test-retest reliability, researchers tested the reliability and validity of scale and created the final BARSHSS-CV.

**Results:**

BARSHSS-CV Cronbach’s α=0.951, content validity = 0.933, test-retest reliability = 0.963 and factor cumulative contribution rate = 67.168% by exploratory factor analysis. By confirmatory factor analysis, Chi square value (χ2) was 442.117, degrees of freedom (df) was 161, Chi square value/degrees of freedom (χ2 /df) was 2.746, root-mean-square error of approximation (RMSEA) was 0.065, goodness of fit index (GFI) was 0.902, incremental fit index (IFI) was 0.955, comparative fit index (CFI) was 0.955, normed fit index (NFI) was 0.931, Tueker-Lewis index (TLI) was 0.947. BARSHSS-CV consisted of 20 items and 3 dimensions.

**Conclusions:**

BARSHSS-CV with good test-retest reliability and content/construct validity is a brief and economical tool for assessing the state of respiratory system amongst adult communities. BARSHSS-CV may help medical staff in community primary medical institutions quickly, conveniently and economically assess the status of respiratory system and the main problems of respiratory system in community adults.

**Electronic supplementary material:**

The online version of this article (10.1186/s12913-018-3505-z) contains supplementary material, which is available to authorized users.

## Background

Globally, the issue of air pollution has impacted the health of millions of individuals. [1]. Air pollution directly contributes to many serious effects on public health in China The National Plan on Air Pollution Control was formulated by the Chinese government in 2012, which set goals and measures to prevent and control air pollution. The first plan was proposed in 2013, which projected that by 2017 the quality of air would be dramatically improved [2]. Infectious diseases are an important threat to human health, and infectious diseases of respiratory system are the most common [3]. Chronic obstructive pulmonary disease (COPD) is an important cause of premature mortality, and asthma prevalence also continues to increase in community adults [4, 5]. Respiratory system diseases are very common diseases contributing to the world’s global disease burden. Collectively respiratory system diseases of adults are associated with a significant burden on healthcare systems, as well as on society [6, 7]. Effective assessment and management plans have been shown to reduce morbidity and mortality from respiratory system diseases among community adults [7]. With the increase of atmospheric concentrations of SO_2_, CO and suspended particles, the variation of pathogenic microorganisms, and the increase of drug-resistant bacteria, the number of people suffering from respiratory system diseases and the mortality of related diseases have increased significantly in the community [3, 8, 9].

At present, much research is focused on the diagnosis and treatment of specific respiratory system diseases in hospitalized patients. However, there are relatively few studies on the respiratory system health level of adults in the community [10, 11]. The evaluation of respiratory system health status in hospitalized patients is usually based on a large number of laboratory examinations and imaging examinations. For example, in blood tests an increase in the number of white blood cells and an increase in the proportion of neutrophils may indicate bacterial infection. In the sputum examination, the drug sensitivity test for bacteria in sputum can guide clinical medication. Chest X-ray examination, CT examination and MRI examination are helpful in the diagnosis of respiratory system organic lesions [3]. However, these medical examinations need to rely on many medical devices and professional technicians. Due to manpower, material resource, financial resource and damage of medical examinations, community-based adults generally can’t receive these medical examinations frequently. Furthermore, it is difficult to be widely and economically used to assess the respiratory health status of adults in the community. Therefore, our research team developed a brief adult respiratory system health status scale-community version (BARSHSS-CV) and tested its reliability and validity. The validity reflects whether the measurement tool effectively measures what the researcher wants to measure. It mainly evaluates the accuracy and correctness of scale. The reliability reflects the measured value variation caused by random errors in the measurement process. It mainly evaluates the accuracy and stability of scale [12]. Potentially the BARSHSS-CV may act as a brief, rapid, effective and economical evaluation tool for extensively assessing the respiratory system health status among community adults in the future.

## Methods

### Development of BARSHSS-CV-I

Using common clinical characteristics and pathogenic factors of the respiratory system diseases as the theoretical basis [3] and through extensive reference to relevant literature, an initial pool of 40 items was generated (Additional file 1). After analyzing and discussing, the research team retained 26 items and developed an initial scale (brief adult respiratory system health status scale-community version-I, BARSHSS-CV-I) (Additional file 2), which included 26 items and 4 dimensions. The four dimensions were named: Dimension 1, mild respiratory system symptom; Dimension 2, severe system respiratory symptom; Dimension 3, medical history; Dimension 4, susceptibility factor. All of the items were presented in simple and reader-friendly language so that the adults in the community could clearly understand the meaning of each item [13, 14].

### Development of BARSHSS-CV-II

The research team invited six related experts from the medical university and the hospital (including two clinical doctors, two clinical nurses and two public health experts) to assess the scale content validity (face validity). 3, on the evaluation standard represented a strong relation, whilst 1 represented no relation. The experts were asked to describe if they felt the item was “strongly related” or “not related” to “adult respiratory system health status” (i.e. the theme of the overall questionnaire). Based on the results of the expert review, 6 items of BARSHSS-CV-Iwere deleted to form BARSHSS-CV-II which included 20 items and 4 dimensions with the unchanged names of each dimension. Three experts rated 1 point (not related) for six items which were deleted. The deleted items were: Q5. I often feel weak; Q6. I often feel powerless; Q7. I often feel my chest uncomfortable when I stay in a hot room for a long time. Q8. I often feel my chest uncomfortable when I stay in a cold room for a long time; Q20. My chest had been traumatized in the past; Q21.I received the surgical treatment of the chest in the past. These experts thought that Q5 and Q6 were common symptoms of many diseases and were not specific symptoms of respiratory system diseases. These experts thought that when a healthy person stayed in a hot or cold room for a long time, the person might also feel uncomfortable. Therefore, Q7 and Q8 were not specific symptoms of respiratory system diseases. These experts thought that chest trauma and chest surgery might damage the muscles and bones of chest, but they did not necessarily damage the respiratory system. Therefore, Q20 and Q21 were also deleted. Finally, this scale retained 20 items (Table 1). Each item was rated according to a 5-point Likert scale (5 = completely agree; 4 = agree most; 3 = moderately agree; 2 = agree a small part, 1 = disagree). In the way of reverse scoring, the scores were 1 point, 2 points, 3 points, 4 points, and 5 points, respectively. The total score of scale was the sum of all items’ scores. The higher the score, the better the community adult respiratory system health status. Subsequently, 5 community adults were asked to complete BARSHSS-CV-II to test scale wording and comprehension so that we could improve item wording and statement expression. Every item in the BARSHSS-CV-IIwas presented in simple and reader-friendly language so that the community adults could clearly understand the meaning of items [13, 14].

**Table 1:** The content of the brief adult respiratory system health status scale-community version (BARSHSS-CV)

Dimensions	Items	Completely agree 5	Agree most 4	Moderately agree 3	Agree a small part 2	Disagree 1
Mild respiratory system symptom	Q1. I often catch a cold.	5	4	3	2	1
	Q2. I often cough.	5	4	3	2	1
	Q3. I often feel phlegm in my throat.	5	4	3	2	1
	Q4. I often feel chest tightness.	5	4	3	2	1
Severe respiratory system symptom	Q5. I often have whooping or whistling sounds when I breathe.	5	4	3	2	1
	Q6. I often have difficulty breathing when I sleep at night.	5	4	3	2	1
	Q7. I often walk slowly due to the dyspnea.	5	4	3	2	1
	Q8. I often have difficulty breathing after I perform mild activity.	5	4	3	2	1
	Q9. I am now suffering from a respiratory system disease.	5	4	3	2	1
	Q10. When I suffer from a respiratory system disease, it takes a long time to recover.	5	4	3	2	1
	Q11. I often cannot work, learn, or carry out outdoor activities due to respiratory system diseases.	5	4	3	2	1
	Q12. I often go to the hospital for examinations and treatments due to respiratory system diseases.	5	4	3	2	1
	Q13. I often use some drugs for the treatment of respiratory system diseases.	5	4	3	2	1
Susceptibility factor	Q14. I often smoke.	5	4	3	2	1
	Q15. My family members often smoke.	5	4	3	2	1
	Q16. My family members often suffered from respiratory system diseases in the past.	5	4	3	2	1
	Q17. I often suffered from respiratory system diseases in the past.	5	4	3	2	1
	Q18. I am often in a haze environment.	5	4	3	2	1
	Q19. My work environment is full of dust or harmful gases.	5	4	3	2	1
	Q20. I am allergic to pollen, dust, animal fur, or some gases.	5	4	3	2	1

### Large sample testing and development of the final BARSHSS-CV

A randomized cluster sampling strategy was used to recruit adults in the communities of Baoding City, Shijiazhuang City, Cangzhou City and Chifeng City in China, between June 2015 and May 2016. Four cities were selected because air pollution in each of these cities is at a serious level [15]. Researchers randomly selected 1 district from each city, a total of 4 districts. Subsequently, 4 communities were respectively randomly selected from these 4 districts. Then, the researchers conducted the questionnaire survey of adults in the 4 communities. The researchers planned to investigate 150 community adults in each community, a total of 600 community adults. Finally, the research team investigated 615 adults in the communities. Inclusion criteria: (1) Community adults that have a satisfactory level of understanding, can correctly understand the contents of questionnaires their respective answers. (2) Not suffering from brain diseases, mental diseases or other serious diseases. Sample size determination: When we evaluated the hypothesized measurement model, the exploratory factor analysis (EFA) and the confirmatory factory analysis (CFA) were appropriately used. 10–15 individuals per item were included in the sample size for the factor analysis. Factor analysis results were relatively more stable and reliable in cases where the sample size is greater than 20 individuals per item [16]. Since this scale contained 20 items, the large sample should be more than 400. However, to increase the results reliability and stability, after comprehensively considering the feasibility of this research, we increased the number of samples. Therefore, finally, a total of 615 questionnaires were distributed. 12 adults did not complete the demographic characteristics questionnaire or this scale. 19 adults did not complete this scale. Finally, 584 complete and valid questionnaires were recovered, and the valid recovery rate was 94.96% (94.96% =584/615). By analyzing the data of 584 valid questionnaires, the researchers tested the scale internal reliability and content/construct validity and developed the final scale (BARSHSS-CV). The test-retest reliability of BARSHSS-CV was tested in the subsample of 50 participants from the large sample over two week’s interval [17]. By asking if the participants had a change in health status during the 2 week interval, researchers determined that the participants had stable health status during the 2 week interval between test and re-test.

### Ethical consideration and survey method

The Hebei province federation of social science circles approved this study (Permit Number: 201501808). This research was conducted according to the standards of the Declaration of Helsinki. The research team explained the purpose of this study to the adults in the communities. After obtaining verbal consent from the community-based adults to participate in the study, researchers provided information to participants on how to complete the questionnaires. The researchers distributed and recovered the questionnaires in the communities. The questionnaires used standardized language and instructions.

### Statistical analysis

The researchers used Epidata3.1 software to input the data twice and completed a consistency check. AMOS 17.0 software and SPSS 17.0 were used to analyze the data. This study used the descriptive statistics (frequency and percentage) to analyze the characteristics of community adults. Testing methods for reliability and validity [12]: ① The confirmatory factor analysis (CFA) and the exploratory factor analysis (EFA) were applied to assess the construct validity of scale. ② The content validity index (CVI) was applied to evaluate the content validity of scale. ③ Mean inter-item correlation coefficient (MIIC) and Cronbach’s α coefficient and test-retest reliability were applied to assess the scale reliability. The level of significance was *p* < 0.05. The retention of factors were based on the following criteria: ① The scree plot of EFA; ② Eigenvalues greater than 1; ③ The factor loadings above 0.500; ④ Items equal to or more than 2 being retained [17]. The model fit of scale structure was considered acceptable if Chi square value/degrees of freedom (χ2 /df) < 3, root-mean-square error of approximation (RMSEA) < 0.08, goodness of fit index (GFI) > 0.90, incremental fit index (IFI) > 0.90, comparative fit index (CFI) > 0.90, normed fit index (NFI) > 0.90, and Tueker-Lewis index (TLI) > 0.90 [14, 17, 18].

## Results

### Characteristics of the large sample

A total of 615 community adults were given questionnaires, 584 valid questionnaires were recovered; the valid recovery rate was 94.96%. These community adults included 258 men (44.2%) and 326 women (55.8%) from urban area (57.4%) and rural area (42.6%). They included Han race (93.8%) and minority (6.2%). These community adults were classified into four age groups including < 20, 20–39, 40–59 and ≥ 60, accounting for 17.1%, 47.4%, 18.5% and 17.0% respectively. These community adults were classified into three monthly income groups including < 800 RMB, 800–1499 RMB and ≥ 1500 RMB, accounting for 24.7%, 40.9% and 34.4% respectively. The characteristics of the large sample data are shown in detail in Table 2.

**Table 2:** Characteristic data of the large sample

Characteristics	Subjects (n)	%
Gender		
Male	258	44.2
Female	326	55.8
Age		
18~	100	17.1
20~	277	47.4
40~	108	18.5
60~	99	17.0
Race		
Han	548	93.8
Minority	36	6.2
Monthly income (RMB)		
< 800	144	24.7
800~	239	40.9
1500~	201	34.4
Education		
Primary school	70	12.0
High school	261	44.7
Junior college	60	10.3
Undergraduate and above	193	33.0
Occupation		
Manual worker	392	67.1
Mental worker	192	32.9
Do you have a religious faith		
No	541	92.6
Yes	43	7.4
Place of residence		
Urban area	335	57.4
Rural area	249	42.6
Physical examination frequency		
Never	186	31.8
Once a few years	188	32.2
Once a year	168	28.8
A few times a year	42	7.2

The characteristic data of the large sample are presented as frequency and percentage

### Testing of reliability and validity

#### Construct validity

##### Exploratory factor analysis (EFA)

**Fig. 1:**
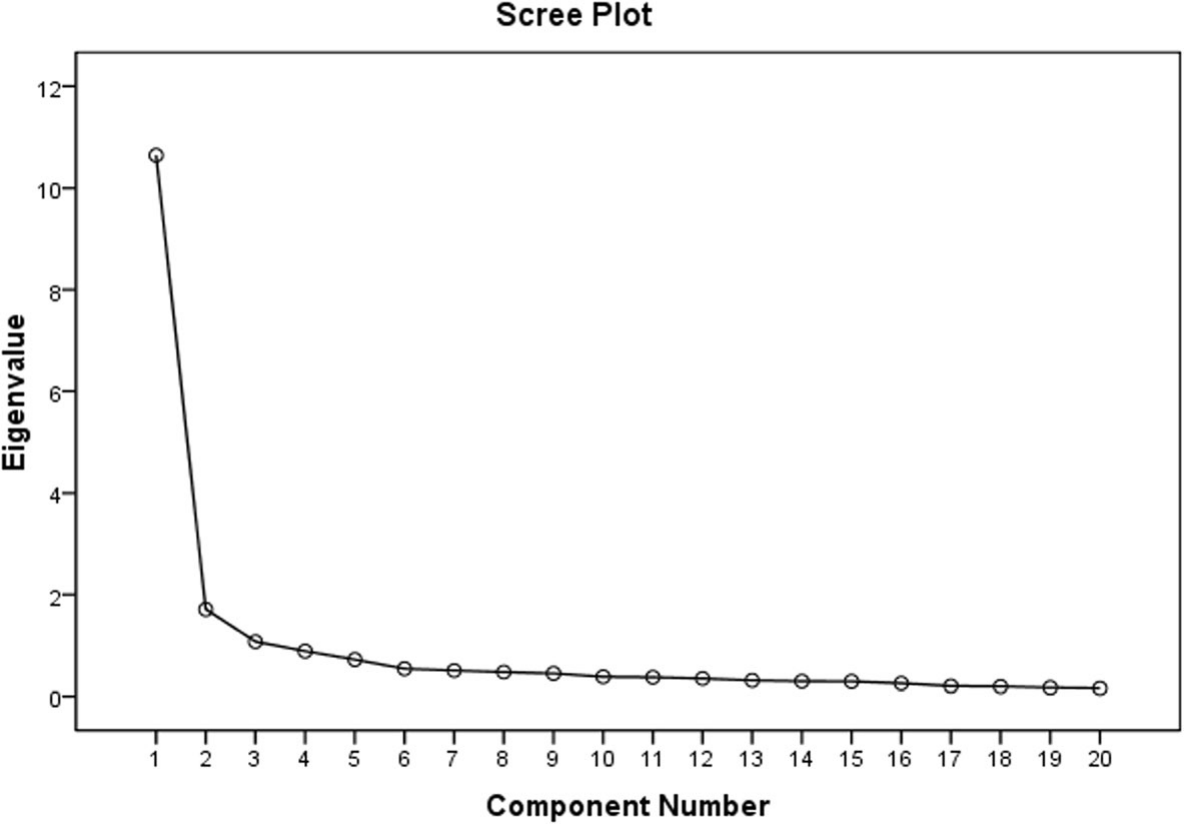
Scree plot of exploratory factor analysis

BARSHSS-CV-II was tested in the 584 participants. To carry out BARSHSS-CV-IIexploratory factor analysis, we used the principal component analysis (PCA) and maximum variance orthogonal rotation methods. The Bartlett sphericity test value was 8705.183 (df = 190, *P* < 0.001) and KMO value (Kaiser-Meyer-Olkin value) was 0.951. From our findings it was clear that the data were suitable for factor analysis. Furthermore, we carried out factor extraction using undefined factor number conditions. The cumulative variance contribution rate of factors was 67.168%. Three factors (Eigenvalue> 1) were extracted. The factor analysis scree plot of BARSHSS-CV-IIindicated an inflection point between the 3rd factor and the 4th factor. The scree plot of EFA indicated the 3-factor structure was suitable (Fig. 1). Through the above comprehensive analysis, the research team thought the final version of BARSHSS-CV contained three factors and 20 items. Final three factors were renamed: Factor 1, mild respiratory system symptom (4 items); Factor 2, severe respiratory system symptom (9 items); Factor 3, susceptibility factor (7 items) (Table 3). The content of the brief adult respiratory system health status scale-community version (BARSHSS-CV) is shown in the Table 1.

**Table 3:** Rotated component matrix, eigenvalue and cumulative variance contribution rate

	Items	Factor 2	Factor 3	Factor 1
	Q1	_	_	0.832
	Q2	_	_	0.849
	Q3	_	_	0.755
	Q4	_	_	0.637
	Q5	0.729	_	_
	Q6	0.741	_	_
	Q7	0.715	_	_
	Q8	0.639	_	_
	Q9	0.729	_	_
	Q10	0.692	_	_
	Q11	0.723	_	_
	Q12	0.761	_	_
	Q13	0.729	_	_
	Q14	_	0.642	_
	Q15	_	0.587	_
	Q16	_	0.750	_
	Q17	_	0.708	_
	Q18	_	0.707	_
	Q19	_	0.740	_
	Q20	_	0.641	_
Eigenvalue		5.729	4.503	3.202
Variance contribution rate (%)		28.643	22.515	16.011
Cumulative variance contribution rate (%)		28.643	51.158	67.168
Factor naming		Severe respiratory system symptom	Susceptibility factor	Mild respiratory system symptom

Factor 1, mild respiratory system symptom; Factor 2, severe system respiratory symptom; Factor 3, susceptibility factor. This symbol ‘_‘indicated that values were less than 0.500. Suppress absolute values less than 0.500

##### Confirmatory factor analysis (CFA)

**Fig. 2:**
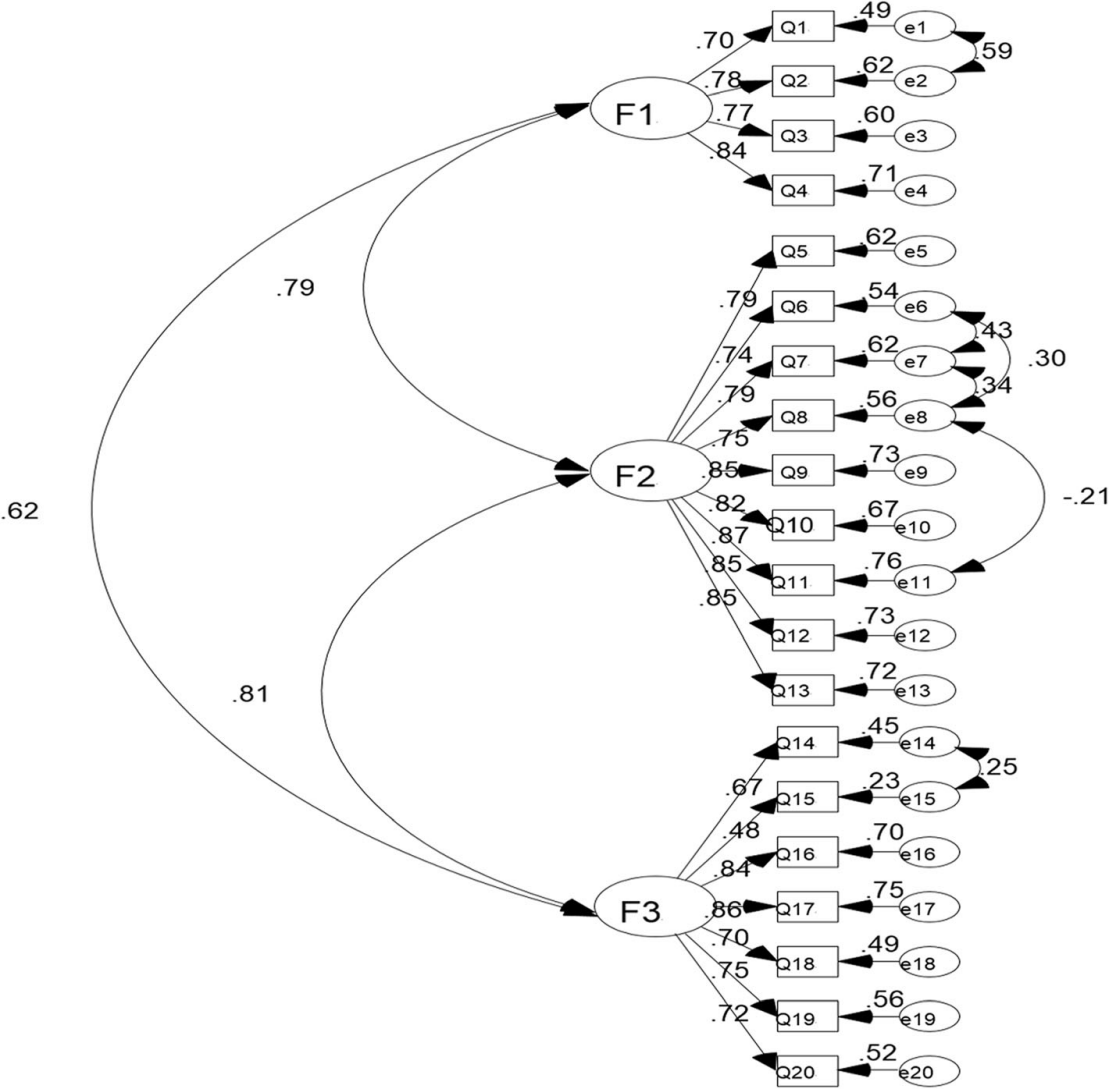
Standard path and parameter estimation of confirmatory factor analysis. F1, Factor 1, mild respiratory system symptom; F2, Factor 2, severe system respiratory symptom; F3, Factor 3, susceptibility factor.

To determine the most appropriate BARSHSS-CV dimensional structure, the researcher used the AMOS17.0 software and randomly selected a 70% sample size of 409 samples and used the maximum likelihood method to perform BARSHSS-CV confirmatory factor analysis of the 20-item and 3-factor structure. The value of Chi square value (χ2) was 442.117. The value of degrees of freedom (df) was 161. The value of Chi square value/degrees of freedom (χ2 /df) was 2.746. The value of root-mean-square error of approximation (RMSEA) was 0.065. The value of goodness of fit index (GFI) was 0.902. The value of incremental fit index (IFI) was 0.955. The value of comparative fit index (CFI) was 0.955. The value of normed fit index (NFI) was 0.931. The value of Tueker-Lewis index (TLI) was 0.947 **(**Table 4). The standard path and parameter estimation of CFA was in the Fig. 2.

**Table 4:** The results of confirmatory factor analysis

*χ* ^*2*^	df	*χ*^*2*^ /df	RMSEA	GFI	IFI	CFI	NFI	TLI
442.117	161	2.746	0.065	0.902	0.955	0.955	0.931	0.947

χ2, Chi square value; df, degrees of freedom; χ2 /df, Chi square value/degrees of freedom; *RMSEA* root-mean-square error of approximation, *GFI* goodness of fit index, *IFI* incremental fit index, *CFI* comparative fit index, *NFI* normed fit index, *TLI* Tueker-Lewis index

#### **Internal** consistency **test**

Correlation coefficients of factors in the BARSHSS-CV ranged from 0.416 to 0.699 (*P* < 0.01). Correlation coefficients between factors and the whole scale of BARSHSS-CV ranged from 0.750 to 0.922 (*P* < 0.01). (Table 5).

**Table 5:** Correlation coefficients among the factors of BARSHSS-CV and between the factors and the total scale of BARSHSS-CV

Factor	Factor 2	Factor 3	BARSHSS-CV
Factor 1	0.591^**^	0.416^**^	0.750^**^
Factor 2	_	0.699^**^	0.922^**^
Factor 3	_	_	0.827^**^

Factor 1, mild respiratory system symptom; Factor 2, severe system respiratory symptom; Factor 3, susceptibility factor. This symbol ‘_‘indicated no such correlation coefficient. ^**^P < 0.01

#### Content validity

Expert evaluation suggests the scale’s content validity index (CVI) was 0.933 and each of the items CVI ranged from 0.667 to 1.00. Five community adults reported that they could clearly understand the meaning of each item without difficulty as soon as we improved item wording and statement expression.

#### Reliability

The Cronbach’s α coefficient of entire BARSHSS-CV was 0.951, and the Cronbach’s α coefficients of all factors were from 0.873 to 0.945. The mean inter-item correlation coefficient value (MIIC) of entire BARSHSS-CV was 0.500. MIIC values of all factors were from 0.511 to 0.656. The entire BARSHSS-CV test-retest reliability coefficient was 0.963, and the test-retest reliability coefficient of each factor were from 0.931 to 0.977 (Table 6).

**Table 6:** MIIC, Cronbach’s α coefficient and test-retest reliability of each factor and the whole BARSHSS-CV

Factor	Number of Items	MIIC	Cronbach’s α	Test-retest reliability
Factor 1	4	0.634	0.873	0.931
Factor 2	9	0.656	0.945	0.944
Factor 3	7	0.511	0.878	0.977
BARSHSS-CV	20	0.500	0.951	0.963

Factor 1, mild respiratory system symptom; Factor 2, severe system respiratory symptom; Factor 3, susceptibility factor. MIIC, mean inter-item correlation coefficients

## Discussion

Worldwide, the older health care model is gradually being transformed; we are shifting from a model that views medical workers as having the leading role in patient care interactions into a newer modern model that involves patients in decisions relating to their health management [19, 20]. The medical policy guidelines and medical system in China mostly focus on the diagnosis, treatment and nursing of the specific respiratory system diseases. The BARSHSS-CV may provide a way to help medical staff in community settings to conveniently and economically assess respiratory system health. Medical staff can also implement targeted intervention and health guidance for the respiratory system health care among community adults to help community adults establish healthy protection awareness, establish a healthy lifestyle, and reduce the incidence of respiratory system diseases to reduce the related medical burden on the government, society, family and themselves individually [21, 22].

Both exploratory factor analysis and confirmatory factor analysis are used when a hypothesized measurement model is assessed in the study. The sample size in the study should be 10–15 subjects per item for factor analysis of scale [23]. The sample size of our research was enough for factor analysis of BARSHSS-CV. In the exploratory factor analysis, to assess the suitability of analysis, Bartlett sphericity test (8705.183, df = 190, *P* < 0.001) was significant and the Kaiser-Meyer-Olkin value (0.951) in our study was greater than 0.6 [24]. The above results showed that the scale data were very suitable for factor analysis. The results of exploratory factor analysis indicated 20 items of BARSHSS-CV loaded substantially onto 3 conceptually clear factors. The dimension 3 (medical history) and the dimension 4 (susceptibility factor) in the BARSHSS-CV-II were merged into dimension 3 (susceptibility factor) in the final BARSHSS-CV. The reason for the change of two dimensions may be because the medical history dimension is also essentially the measurement of the susceptibility of the individual’s respiratory system. If individuals suffered from related respiratory system diseases in the past, it may indicate that individuals may have some genetic or environmental susceptibility factors, and the probability of recurring respiratory system adverse symptoms may increase correspondingly. Therefore, the research team finally merged the medical history dimension into the susceptibility factor dimension so that the BARSHSS-CV structure becomes more concise, clear and easier to use. In the confirmatory factor analysis, the model goodness of fit is assessed by RMSEA < 0.080, GFI > 0.900, IFI > 0.900, CFI > 0.900, NFI > 0.900, TLI > 0.900 [14, 17, 18]. The results of confirmatory factor analysis in our research met these criteria. The results of confirmatory factor analysis indicate the stability and fit of 3-factor model structure of BARSHSS-CV are both good.

In the internal consistency test of BARSHSS-CV, correlation coefficients between all factors and the whole BARSHSS-CV are from 0.750 to 0.922, showing that the internal consistency of BARSHSS-CV is good. The values of correlation coefficients between all factors are from 0.416 to 0.699, showing that the correlation of the factors is a moderate correlation [14]. Thus, the results of BARSHSS-CV show that there is a moderate correlation among different factors. But there are also a certain degree of differences between different factors. Therefore, these factors can measure different aspects of community adult respiratory system health status. Three factors can effectively and comprehensively measure the respiratory system health status of adults in the community.

If items pertaining to the scale can identify the content and topic that we intend to measure, this is referred to as the content validity. The number of expert choices of 3 and 2, which is divided by the total number of experts, represents each item content validity index belonging to the scale. All item content validity indexes make up the average total content validity index [14]. The content validity index of scale was 0.933. The content validity indexes of all items were from 0.667 to 1.00. The results indicates that BARSHSS-CV can measure the variables we want to measure; all items can reflect the correct content, and the content validity of BARSHSS-CV is very good.

Mean inter-item correlation (MIIC), Cronbach’s alpha and test-retest reliability can be used to evaluate the reliability of scale [17, 25, 26]. When the MIIC of scale is greater than 0.3, the internal consistency of scale is usually acceptable [26]. The mean inter-item correlation coefficient values of the whole BARSHSS-CV and all factors were greater than 0.5. The usual criterion of a satisfactory internal consistency is a Cronbach’s alpha of ≥0.7 [14]. In our research, the Cronbach’s alpha value of whole BARSHSS-CV and the Cronbach’s alpha values of all factors were greater than 0.8. BARSHSS-CV also reveals a high test-retest reliability over a two week interval, with test-retest reliability coefficients across all dimensions and the whole BARSHSS-CV ranging from 0.931 to 0.977. Therefore, after the comprehensive analysis, BARSHSS-CV developed in our research has a satisfactory reliability.

## Limitations and future direction

Because of limited study conditions, the scope of sampling needs to be further expanded making BARSHSS-CV more widely verified and applied in more areas of country. BARSHSS-CV can be further improved and revised in the future. A major limitation of this study, and an area for future research should be to investigate the concurrent validity of this new measure with other established questionnaire measures of respiratory health status. Using laboratory examinations and imaging examinations as the measurement golden criterion of the respiratory system health status of adults in the community, it requires a high cost and medical resources. Furthermore, the compliance of participants will also significantly decline. Due to the lack of an economical, effective, and appropriate golden criterion for measuring the respiratory system health status of adults in the community, testing the criterion validity of BARSHSS-CV is difficult [17]. In the future study, we will recruit some patients in the respiratory departments of hospitals and compare the BARSHSS-CV score with the results of laboratory examinations and imaging examinations of the patients to further evaluate the sensitivity and specificity of BARSHSS-CV, so that BARSHSS-CV can be more helpful and better for clinical decision. Furthermore, the scale validation in other countries and the cross-cultural scale revision are needed to develop the international application of BARSHSS-CV in the future.

## Conclusion

To sum up, the research has rigorously developed and validated BARSHSS-CV with good internal/re-test reliability and good content/construct validity. BARSHSS-CV is a brief, rapid, effective and economical evaluation tool for extensively assessing the respiratory system health status among community adults in the future. BARSHSS-CV can be used to assess the respiratory system health status of adults in the community, help medical staff in health care institutions conduct targeted interventions, as well as provide health guidance for community adults relating to the health care of the respiratory system and help community adults establish a healthy lifestyle.

## Additional files

Additional file 1: The item pool. The initial pool of 40 items. (DOCX 14 kb)

Additional file 2: BARSHSS-CV-I. The content of the brief adult respiratory system health status scale-community version-I(BARSHSS-CV-I). (DOCX 16 kb)

## Abbreviations

BARSHSS-CV: Brief adult respiratory system health status scale-community version
CFA: Confirmatory factor analysis
CFI: Comparative fit index
CVI: Content validity index
df: Degrees of freedom
EFA: Exploratory factor analysis
GFI: Goodness of fit index
IFI: Incremental fit index
KMO: Kaiser-Meyer-Olkin
MIIC: Mean inter-item correlation coefficients
NFI: Normed fit index
PCA: Principal component analysis
RMSEA: Root-mean-square error of approximation
TLI: Tueker-Lewis index
χ2 /df: Chi square value/degrees of freedom
χ2: Chi square value
