# Supplementary material for: Brief adult respiratory system health status scale-community version (BARSHSS-CV): developing and evaluating the reliability and validity
Source: BMC Health Serv Res. 2018 Sep 3;18:683. doi: 10.1186/s12913-018-3505-z (PMC6122650; doi:10.1186/s12913-018-3505-z)
Supplement: Supplementary file 1 — The item pool. The initial pool of 40 items. (DOCX 14 kb) [file 12913_2018_3505_MOESM1_ESM.docx]

The pool of 40 items in Additional file 1

| **Number** | **Items** |
| --- | --- |
| Q1. | I often catch a cold. |
| Q2. | I often have a fever. |
| Q3. | I often feel a whole body chill. |
| Q4. | I often cough. |
| Q5. | I often feel phlegm in my throat. |
| Q6. | I often feel weak. |
| Q7. | I often feel powerless. |
| Q8. | I often feel my chest uncomfortable when I stay in a hot room for a long time. |
| Q9. | I often feel my chest uncomfortable when I stay in a cold room for a long time. |
| Q10. | I often feel uncomfortable with the respiratory system in the hot summer. |
| Q11. | I often feel uncomfortable with the respiratory system in the cold winter. |
| Q12. | I often feel uncomfortable with the respiratory system at high altitude. |
| Q13. | I often feel short of breath at high altitude. |
| Q14. | I often feel uncomfortable with the respiratory system in a low oxygen environment. |
| Q15. | I often feel short of breath in a low oxygen environment. |
| Q16. | I often feel chest tightness. |
| Q17. | I often feel a chest pain. |
| Q18. | I often feel uncomfortable in the chest. |
| Q19. | I often have whooping or whistling sounds when I breathe. |
| Q20. | I often have difficulty breathing when I sleep at night. |
| Q21. | I often walk slowly due to the dyspnea. |
| Q22. | I often have difficulty breathing after I perform mild activity. |
| Q23. | I am now suffering from a respiratory system disease. |
| Q24. | When I suffer from a respiratory system disease, it takes a long time to recover. |
| Q25. | I often cannot work, learn, or carry out outdoor activities due to respiratory system diseases. |
| Q26. | I often go to the hospital for examinations and treatments due to respiratory system diseases. |
| Q27. | I often use some drugs for the treatment of respiratory system diseases. |
| Q28. | I often smoke. |
| Q29. | My family members often smoke. |
| Q30. | My family members often suffered from respiratory system diseases in the past. |
| Q31. | My father often suffered from respiratory system diseases in the past. |
| Q32. | My mother often suffered from respiratory system diseases in the past. |
| Q33. | My brothers often suffered from respiratory system diseases in the past. |
| Q34. | My sisters often suffered from respiratory system diseases in the past. |
| Q35. | I often suffered from respiratory system diseases in the past. |
| Q36. | My chest had been traumatized in the past. |
| Q37. | I received the surgical treatment of chest in the past. |
| Q38. | I am often in a haze environment. |
| Q39. | My work environment is full of dust or harmful gases. |
| Q40. | I am allergic to pollen, dust, animal fur, or some gases. |
