# Supplementary material for: Brief adult respiratory system health status scale-community version (BARSHSS-CV): developing and evaluating the reliability and validity
Source: BMC Health Serv Res. 2018 Sep 3;18:683. doi: 10.1186/s12913-018-3505-z (PMC6122650; doi:10.1186/s12913-018-3505-z)
Supplement: Supplementary file 2 — BARSHSS-CV-I. The content of the brief adult respiratory system health status scale-community version-I(BARSHSS-CV-I). (DOCX 16 kb) [file 12913_2018_3505_MOESM2_ESM.docx]

The content of the brief adult respiratory system health status scale-community version-Ⅰ (BARSHSS-CV-Ⅰ) in Additional file 2

| **Dimensions** | **Items** | **Completely agree**  **5** | **Agree most**  **4** | **Moderately agree**  **3** | **Agree a small part**  **2** | **Disagree**  **1** |
| --- | --- | --- | --- | --- | --- | --- |
| **Mild respiratory system symptom** | Q1. I often catch a cold. | 5 | 4 | 3 | 2 | 1 |
|  | Q2. I often cough. | 5 | 4 | 3 | 2 | 1 |
|  | Q3. I often feel phlegm in my throat. | 5 | 4 | 3 | 2 | 1 |
|  | Q4. I often feel chest tightness. | 5 | 4 | 3 | 2 | 1 |
|  | Q5. I often feel weak. | 5 | 4 | 3 | 2 | 1 |
|  | Q6. I often feel powerless. | 5 | 4 | 3 | 2 | 1 |
|  | Q7. I often feel my chest uncomfortable when I stay in a hot room for a long time. | 5 | 4 | 3 | 2 | 1 |
|  | Q8. I often feel my chest uncomfortable when I stay in a cold room for a long time. | 5 | 4 | 3 | 2 | 1 |
| **Severe respiratory system symptom** | Q9. I often have whooping or whistling sounds when I breathe. | 5 | 4 | 3 | 2 | 1 |
|  | Q10. I often have difficulty breathing when I sleep at night. | 5 | 4 | 3 | 2 | 1 |
|  | Q11. I often walk slowly due to the dyspnea. | 5 | 4 | 3 | 2 | 1 |
|  | Q12. I often have difficulty breathing after I perform mild activity. | 5 | 4 | 3 | 2 | 1 |
|  | Q13. I am now suffering from a respiratory system disease. | 5 | 4 | 3 | 2 | 1 |
|  | Q14. When I suffer from a respiratory system disease, it takes a long time to recover. | 5 | 4 | 3 | 2 | 1 |
|  | Q15. I often cannot work, learn, or carry out outdoor activities due to respiratory system diseases. | 5 | 4 | 3 | 2 | 1 |
|  | Q16. I often go to the hospital for examinations and treatments due to respiratory system diseases. | 5 | 4 | 3 | 2 | 1 |
|  | Q17. I often use some drugs for the treatment of respiratory system diseases. | 5 | 4 | 3 | 2 | 1 |
| **Medical history** | Q18. My family members often suffered from respiratory system diseases in the past. | 5 | 4 | 3 | 2 | 1 |
|  | Q19. I often suffered from respiratory system diseases in the past. | 5 | 4 | 3 | 2 | 1 |
|  | Q20. My chest had been traumatized in the past. | 5 | 4 | 3 | 2 | 1 |
|  | Q21.I received the surgical treatment of the chest in the past. | 5 | 4 | 3 | 2 | 1 |
| **Susceptibility factor** | Q22. I often smoke. | 5 | 4 | 3 | 2 | 1 |
|  | Q23. My family members often smoke. | 5 | 4 | 3 | 2 | 1 |
|  | Q24. I am often in a haze environment. | 5 | 4 | 3 | 2 | 1 |
|  | Q25. My work environment is full of dust or harmful gases. | 5 | 4 | 3 | 2 | 1 |
|  | Q26. I am allergic to pollen, dust, animal fur, or some gases. | 5 | 4 | 3 | 2 | 1 |
